# Crystal structures of N-myristoylated lipopeptide-bound HLA class I complexes indicate reorganization of B-pocket architecture upon ligand binding

**DOI:** 10.1016/j.jbc.2022.102100

**Published:** 2022-06-03

**Authors:** Minori Asa, Daisuke Morita, Jin Kuroha, Tatsuaki Mizutani, Naoki Mori, Bunzo Mikami, Masahiko Sugita

**Affiliations:** 1 Laboratory of Cell Regulation, Institute for Life and Medical Sciences, Kyoto University, Kyoto, Japan; 2 Laboratory of Cell Regulation and Molecular Network, Graduate School of Biostudies, Kyoto University, Kyoto, Japan; 3 Laboratory of Chemical Ecology, Division of Applied Life Sciences, Graduate School of Agriculture, Kyoto University, Kyoto, Japan; 4 Laboratory of Applied Structural Biology, Division of Applied Life Sciences, Graduate School of Agriculture, Kyoto University, Kyoto, Japan

**Keywords:** antigen presentation, crystal structure, human, immunology, protein myristoylation, β2m, β2-microglobulin, CTL, cytotoxic T-lymphocyte, HLA, human leukocyte antigen, Myr-GANF, N-myristoylated Gly-Ala-Asn-Phe, TCR, T-cell receptor, VDW, Van der Waals

## Abstract

Rhesus monkeys have evolved MHC-encoded class I allomorphs such as Mamu-B∗098 that are capable of binding N-myristoylated short lipopeptides rather than conventional long peptides; however, it remains unknown whether such antigen-binding molecules exist in other species, including humans. We herein demonstrate that human leukocyte antigen (HLA)-A∗24:02 and HLA-C∗14:02 proteins, which are known to bind conventional long peptides, also have the potential to bind N-myristoylated short lipopeptides. These HLA class I molecules shared a serine at position 9 (Ser9) with Mamu-B∗098, in contrast to most MHC class I molecules that harbor a larger amino acid residue, such as tyrosine, at this position. High resolution X-ray crystallographic analyses of lipopeptide-bound HLA-A∗24:02 and HLA-C∗14:02 complexes indicated that Ser9 was at the bottom of the B pocket with its small hydroxymethyl side chain directed away from the B-pocket cavity, thereby contributing to the formation of a deep hydrophobic cavity suitable for accommodating the long-chain fatty acid moiety of lipopeptide ligands. Upon peptide binding, however, we found the hydrogen-bond network involving Ser9 was reorganized, and the remodeled B pocket was able to capture the second amino acid residue (P2) of peptide ligands. Apart from the B pocket, virtually no marked alterations were observed for the A and F pockets upon peptide and lipopeptide binding. Thus, we concluded that the structural flexibility of the large B pocket of HLA-A∗2402 and HLA-C∗1402 primarily accounted for their previously unrecognized capacity to bind such chemically distinct ligands as conventional peptides and N-myristoylated lipopeptides.

MHC class I molecules bind peptide antigens and present them to cytotoxic T lymphocytes (CTLs) (1). In virus-infected cells, peptide fragments derived from cytosolic viral proteins are transported into the lumen of the endoplasmic reticulum, in which unstable MHC class I heavy chain–β2-microglobulin (β2m) heterodimer complexes are stabilized by binding peptides of 8 to 11 aa in length (2, 3). The peptide-bound MHC class I trimer complexes exit the endoplasmic reticulum and reach the cell surface to display viral peptides in the context of MHC class I molecules, thereby helping CTLs to detect virus-infected cells and eliminate them (4). The basic principle of MHC class I–mediated peptide antigen presentation is recognized as one of the major paradigms that modern immunology has established; however, there may be some scope for further consideration because a distinct subset of MHC class I molecules has recently been identified in rhesus monkeys that bind lipidated short peptides (lipopeptides) rather than conventional long peptides (5, 6).

Some viral proteins with the N-terminal Gly-X-X-X-Ser/Thr motif undergo N-myristoylation, a lipid modification of proteins in which the exposed Gly residue is conjugated covalently with a 14-carbon fatty acid (myristic acid) (7). The simian immunodeficiency virus Nef protein, for example, contains the Gly-Gly-Ala-Ile-Ser sequence, and N-myristoylation serves to dictate its pathological function (8, 9). On the other hand, simian immunodeficiency virus–infected monkeys mount CTL responses directed against N-terminal lipopeptide fragments of the N-myristoylated Nef protein (10, 11). Five-mer and 4-mer lipopeptides are captured by the rhesus MHC class I allomorphs, Mamu-B∗098 (5) and Mamu-B∗05,104 (6), respectively, and recognized by specific CTLs. Similar to other MHC class I molecules, the basic six-pocket structure (A through F pockets) is constructed in the antigen-binding groove of Mamu-B∗098 and Mamu-B∗05104, and the B and F pockets function critically in capturing anchor components of ligands. The large B pockets of Mamu-B∗098 and Mamu-B∗05104 are capable of accommodating the long-chain fatty acid moiety of lipopeptide ligands by establishing numerous Van der Waals (VDW) interactions between the pocket surface and bound acyl chain, contrasting sharply with those of other MHC class I molecules that accommodate the P2 amino acid residue of peptide ligands (5, 6). On the other hand, F pockets that bind the C-terminal amino acid residue of either peptide or lipopeptide ligands are virtually identical in structure and function. These lines of evidence suggest that the architecture of B pockets may be critical to peptide and lipopeptide binding.

MHC class I allomorphs that bind lipopeptides have been identified so far only in rhesus monkeys, and it remains to be determined whether such molecules exist in other species, including humans. In the present study, we focused on a particular amino acid residue at position 9, which may function as a critical element for determining the size and chemical properties of the B pocket, and found that human leukocyte antigen (HLA)-A∗24:02 and HLA-C∗14:02 molecules have the potential to bind N-myristoylated lipopeptides besides conventional peptides. The X-ray crystallographic structures of peptide- and lipopeptide-bound HLA-A∗24:02 and HLA-C∗14:02 complexes detected B-pocket remodeling mechanisms, which explained how these molecules could bind chemically distinct ligands.

## Results

### Lipopeptide ligand-dependent complex formation occurred efficiently for HLA-A∗24:02 but not HLA-A∗24:50

Among members of the classical MHC class I family, the two rhesus lipopeptide-presenting MHC class I allomorphs, Mamu-B∗098 and Mamu-B∗05104, are marked by their exceptionally large B pockets. The depth of the B pocket is determined critically by the amino acid residue at position 9, which is located on the β-sheet floor with its side chain protruding upward into the lumen of the B pocket (12). Mamu-B∗098 and Mamu-B∗05104 have serine (Ser9) and glycine (Gly9) at this position, respectively, and their crystallographic structures point to a critical role for these small amino acid residues in sustaining the structure of the B pocket cavity suitable for accommodating the long-chain fatty acid moiety of lipopeptide ligands (5, 6). In humans, tyrosine (Tyr9) is regarded as the consensus residue for this position (13); however, a fraction of HLA class I allomorphs expresses Ser9 as is the case with Mamu-B∗098, leading us to the hypothesis that Ser9-containing HLA class I allomorphs may include those with the ability to bind lipopeptides. In this respect, we directed our attention to a pair of mutually related allomorphs, HLA-A∗24:02 and HLA-A∗24:50, which possess Ser9 and Tyr9, respectively, while all the other amino acid residues are identical. We performed side-by-side analyses of these allomorphs to assess the impact of Ser9/Tyr9 exchange on ligand binding.

**Figure 1:**
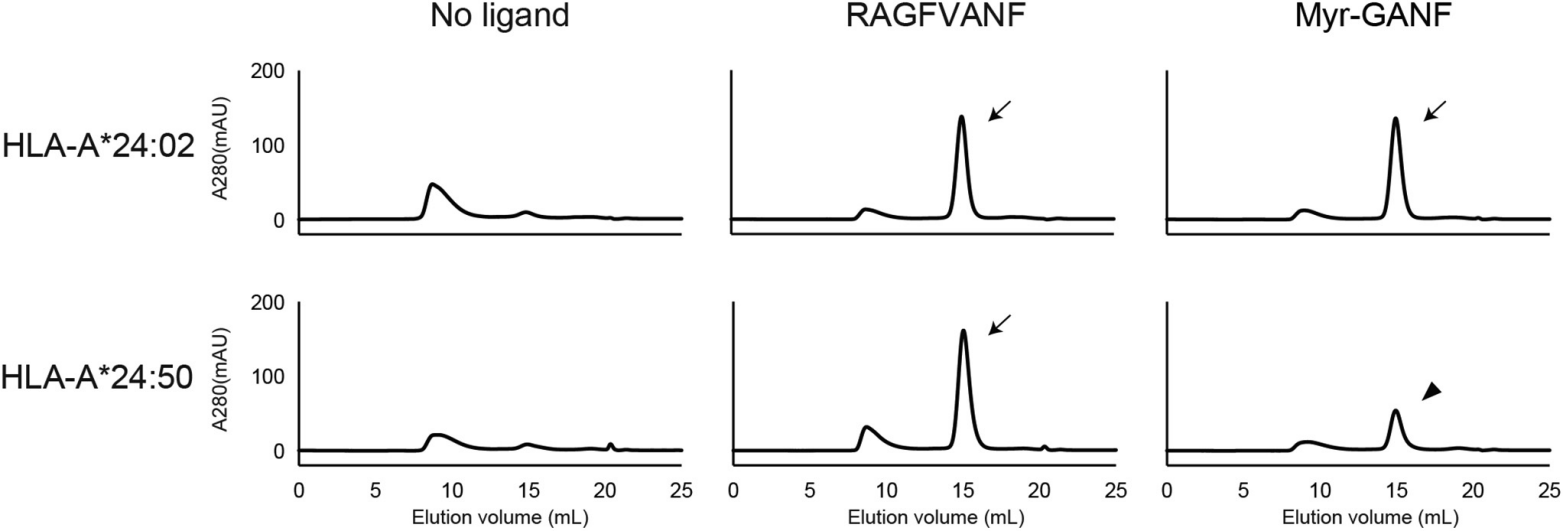
**Ligand-induced assembly of HLA-A∗24:02 and HLA-A∗24:50 molecules.** Assembly of HLA-A∗24:02 heavy chains (*upper panels*) with β2m was induced in a buffer system in the presence of either RAGFVANF (*middle*) or Myr-GANF (*right*) or in their absence (*left*), and the formation of ligand-bound HLA-A∗24:02 complexes was monitored by size-exclusion chromatography (indicated with *arrows*). HLA-A∗24:50 heavy chains were also analyzed in parallel (*lower panels*). Note that complex formation of HLA-A∗24:50 was induced only inefficiently by Myr-GANF (*lower right panel*, indicated with an *arrowhead*). β2m, β2-microglobulin; HLA, human leukocyte antigen.

We first employed an *in vitro* refolding assay in which the soluble form of MHC class I heavy chains was induced to assemble with β2m in a buffer containing appropriate ligands (5). When HLA-A∗24:02 heavy chains and β2m were incubated in the presence of the synthetic peptide, RAGFVANF, stable trimer complexes of the heavy chain, β2m, and ligands were formed, which was monitored by a sharp increase in the absorbance value of *A*_280_ at an elution volume of 15 ml (Fig. 1, *upper middle panel*, indicated with an *arrow*), whereas the corresponding signal was virtually undetectable when the incubation was performed in its absence (*upper left panel*). Peptide-induced complex formation was also induced for HLA-A∗24:50 heavy chains (*lower middle panel*, indicated with an *arrow*), confirming that the refolding capacity *per se* was comparable between HLA-A∗24:02 and HLA-A∗24:50. The synthetic lipopeptide, N-myristoylated Gly-Ala-Asn-Phe (Myr-GANF; note that the C-terminal 3 amino acid residues were identical to the peptide used above), was also able to promote HLA-A∗24:02 complex formation, as evidenced by the increased signal at an elution volume of 15 ml (*upper right panel*, indicated with an *arrow*), which raised the possibility that the Myr-GANF lipopeptide may function as a potent ligand for HLA-A∗24:02. In sharp contrast, Myr-GANF lipopeptide–induced complex formation was reduced markedly for HLA-A∗24:50 (*lower right panel*, indicated with an *arrowhead*) as compared with HLA-A∗24:02 (*upper right panel*). Taken together, these results suggest that the Ser9-containing HLA-A∗24:02 allomorph may have evolved a previously unrecognized ability to bind not only conventional peptides but also N-myristoylated lipopeptides.

### The Myr-GANF lipopeptide occupied the antigen-binding groove of HLA-A∗24:02

**Figure 2:**
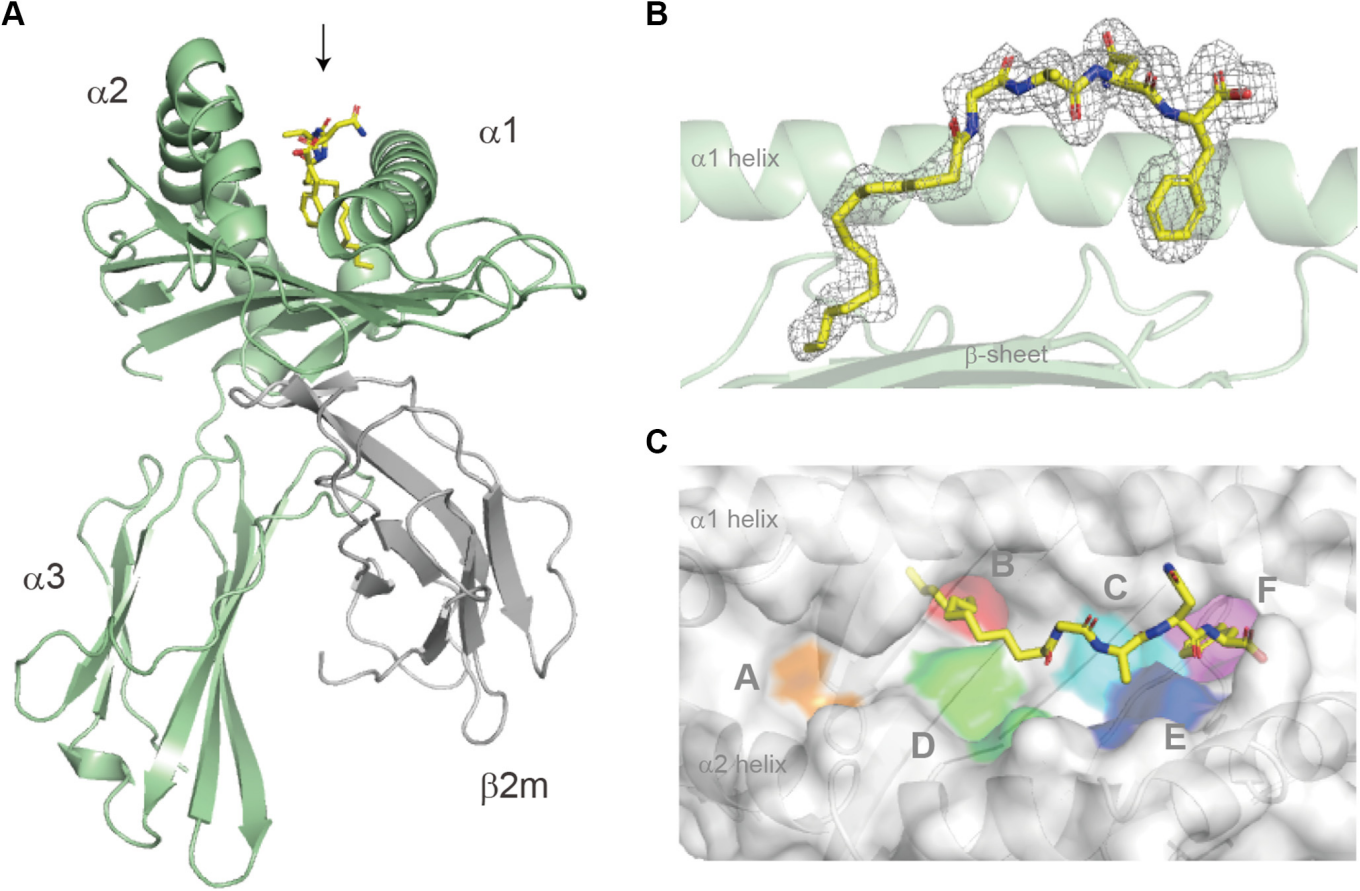
**The overall structure of the HLA-A∗24:02:Myr-GANF complex.***A*, a side view of the trimer complex composed of the ectodomain of HLA-A∗24:02 heavy chains (*green*), β2m (*gray*), and Myr-GANF (*yellow sticks*, indicated with an *arrow*) is shown. *B*, the binding of Myr-GANF (*yellow sticks*) to HLA-A∗24:02 (*green*) is demonstrated by phenix polder omit map (*gray mesh*) contoured at 3.5σ. *C*, a top view of the surface of the antigen-binding groove with pockets A through F is shown. Note that the lipopeptide ligand (*yellow sticks*) was accommodated in the antigen-binding groove constructed between the α1 and α2 helices. β2m, β2-microglobulin; HLA, human leukocyte antigen; Myr-GANF, N-myristoylated Gly-Ala-Asn-Phe.

**Figure 3:**
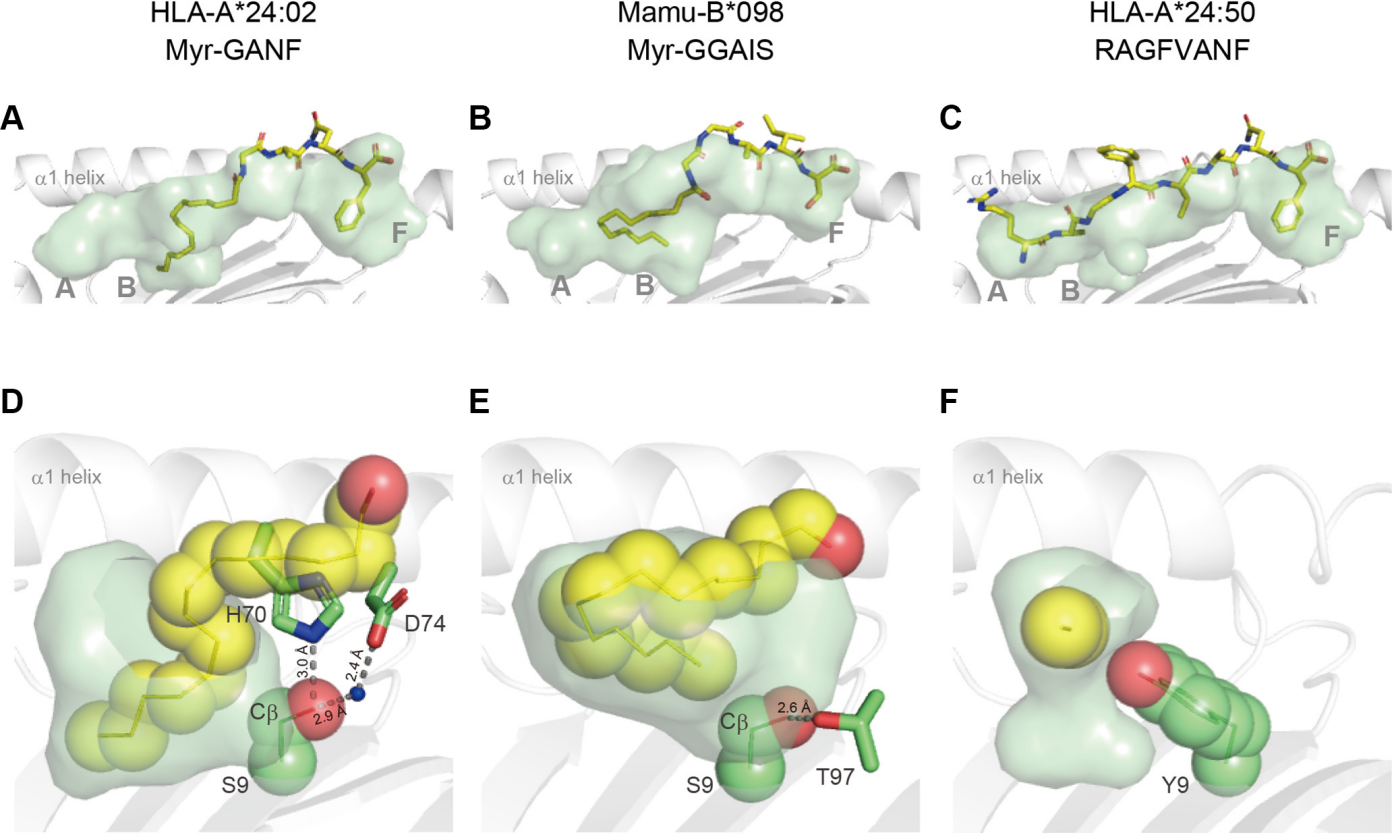
**Accommodation of the myristoyl group of Myr-GANF in the B pocket of HLA-A∗24:02.** Lipopeptide ligands (*yellow sticks*) captured in the semitransparent antigen-binding groove are shown for the HLA-A∗24:02:Myr-GANF *(A)* and Mamu-B∗098:Myr-GGAIS *(B)* complexes. The myristoyl group of each lipopeptide ligand is displayed in *yellow* as a space-filling model with the semitransparent B pocket of HLA-A∗24:02 *(D)* and Mamu-B∗098 *(E)* with an emphasis on the spatial orientation of the side chain of Ser9 (displayed in *green* as a space-filling model) and its hydrogen-bond interactions (*dashed lines*) with side chains of neighboring amino acid residues (*green sticks*). For comparison, the RAGFVANF peptide (*yellow sticks*) captured in the antigen-binding groove of HLA-A∗24:50 *(C)* and the P2 alanine residue (displayed in *yellow* as a space-filling model) accommodated in its B pocket *(F)* are also shown. Note that, unlike Ser9 of HLA-A∗24:02 *(D)* and Mamu-B∗098 *(E)*, the side chain of Tyr9 (depicted in *green* as a pace-filling model) of HLA-A∗24:50 was directed toward the B-pocket cavity *(F)*. HLA, human leukocyte antigen; Myr-GANF, N-myristoylated Gly-Ala-Asn-Phe.

To establish the molecular basis underlying the potential of HLA-A∗24:02 to bind lipopeptides and determine how the amino acid residue at position 9 may impact ligand binding, the soluble form of HLA-A∗24:02 complexed with the Myr-GANF lipopeptide was prepared, and its crystal structure was determined (Table 1). The overall structure of the Myr-GANF–bound HLA-A∗24:02 complex was almost indistinguishable from those of other HLA class I molecules, characterized by semisymmetrical α1 and α2 domains forming an antiparallel β-sheet topped by semiparallel α-helices (Fig. 2*A*). Electron density corresponding to the Myr-GANF lipopeptide was observed in the antigen-binding groove, located between α1 and α2 helices on top of the β-sheet (Fig. 2, *B* and *C*), which provided structural evidence that the lipopeptide served as an authentic ligand for HLA-A∗24:02. We found that molecular elements located at either end of the Myr-GANF lipopeptide, namely the myristoyl group and C-terminal Phe4 residue, interacted with the B and F pockets, respectively, to function as primary anchors, whereas the A pocket was left unoccupied (Fig. 2*C*; also see Fig. 3*A*). These structural features of the Myr-GANF–bound HLA-A∗24:02 complex coincided with the basic principle of lipopeptide binding noted previously for rhesus Mamu-B∗098 and Mamu-B∗05104 (5, 6).

**Table 1:** Data collection and refinement statistics (Molecular replacement)^a^

	HLA-A^∗^24:02 Myr-GANF complex	HLA-A^∗^24:02 RF8 complex	HLA-A^∗^24:50 RAGFVANF complex	HLA-C^∗^14:02 Myr-GAAL complex	HLA-C^∗^14:02 LL8 complex
**PDB ID**	7WT3	7WT4	7WT5	7WJ3	7WJ2
*Data collection*					
Detector at BL26B1	Eiger4M	Eiger4M	Eiger4M	MX225HE	MX225HE
Space group	*P2*_*1*_	*P2*_*1*_	*P2*_*1*_	*C*2	*C*2
Cell dimensions					
a, b, c (Å) β (°)	87.6, 46.7, 143.6 104.9	86.4, 46.6, 141.9 104.0	86.4, 46.6, 141.9 104.0	91.1, 77.5, 60.6 120.9	92.7, 76.9, 62.1 119.5
Trimer in asym. unit	2	2	2	1	1
Resolution (Å)	50–1.89 (2.00–1.89)^a^	50–1.89 (2.01–1.89)	50–2.10 (2.22–2.10)	50–1.56 (1.59–1.56)	50–1.28 (1.30–1.28)
R_merge_	0.057 (0.448)	0.078 (0.472)	0.086 (0.463)	0.044 (0.390)	0.056 (0.384)
I/σ(I)	14.4 (2.83)	11.6 (2.25)	12.6 (2.98)	49.5 (4.80)	36.6 (3.08)
Completeness (%)	99.6 (99.5)	97.6 (97.3)	99.5 (99.1)	99.2 (84.9)	99.0 (97.3)
Redundancy	3.7 (3.9)	2.9 (2.8)	3.8 (3.9)	7.4 (6.2)	4.3 (3.9)
CC(1/2) (%)	99.9 (87.0)	99.7 (79.5)	99.7 (85.7)	99.8 (93.3)	99.6 (91.8)
Wilson B (Å^2^)	27.1	21.1	25.8	16.9	13.6
*Refinement*					
Resolution (Å)	1.89 (1.91–1.89)	1.89 (1.92–1.89)	2.10 (2.13–2.10)	1.56 (1.59–1.56)	1.28 (1.29–1.28)
No. of reflections	89,123 (2904)	85,071 (2706)	64,481 (2715)	50,956 (2725)	96,238 (3046)
*R*_work_/*R*_free_ (%)	20.0 (31.3)/23.5 (33.0)	20.0 (31.1)/23.2 (34.1)	18.6 (23.2)/22.8 (28.4)	17.0 (18.3)/18.8 (21.4)	15.6 (22.9)/17.8 (27.4)
Coordinate error (Å)	0.22	0.22	0.25	0.13	0.13
No. of residues					
Protein	677	715	733	379	384
MYR/EDO/ACT/TRIS/ZN/NA	2/37/3/1/3/0	0/46/1/2/3/0	0/47/0/1/3/0	1/18/0/0/0/0	0/7/1/0/0/1
Water	403	423	334	227	334
*B*-factors (Å^2^)					
Protein	36.8	32.1	37.2	21.3	19.6
Ligand and ion	42.7	36.5	41.6	31.6	26.0
Water	37.5	30.2	32.3	27.8	27.4
RMSDs					
Bond lengths (Å)	0.008	0.008	0.009	0.009	0.008
Bond angles (º)	1.243	1.020	1.094	0.917	1.06
Ramachandran plot					
Favored (%)	98.4	98.6	98.0	98.2	98.4
Outliers (%)	0	0	0	0	0
Crash score	4.41	4.19	5.10	1.41	4.37
Rotamer outliers (%)	1.00	0.95	1.54	0.59	1.40

Abbreviation: PDB, Protein Data Bank.

a The highest resolution shell is shown in parentheses.

### The B-pocket Ser9 residue of HLA-A∗24:02 supported accommodation of the long-chain fatty acid, whereas Tyr9 of HLA-A∗24:50 was likely to generate steric hindrance

The myristoyl group of Myr-GANF fitted in the B pocket of HLA-A∗24:02 in a sigmoid-shaped configuration (Fig. 3, *A* and *D*), which contrasted with the U-shaped packing pattern observed for Mamu-B∗098 (Fig. 3, *B* and *E*). Nevertheless, the spatial orientation of the side chain of Ser9 as well as the role of its β carbon atom (Cβ) in producing VDW forces appeared to be shared between the two molecules. In both cases, Ser9 was located at the bottom of the B pocket, and the hydroxyl group of its side chain was positioned away from the ligand by establishing hydrogen-bond interactions with neighboring amino acid residues (His70 and Asp74 for HLA-A∗24:02 and Thr97 for Mamu-B∗098) (Fig. 3, *D* and *E*), which enabled the Cβ atom of Ser9 to interact with the myristoyl group *via* VDW forces. Therefore, the spatial orientation of the side chain of Ser9 contributed not only to the space expansion of the B pocket cavity but also to the creation of the hydrophobic surface suitable for accommodating the acyl chain of lipopeptide ligands. We considered that this organized structure may not be achieved efficiently in Tyr9-containing MHC class I molecules due to space constraints. Indeed, a crystallographic analysis of HLA-A∗24:50 complexed with the RAGFVANF peptide (Table 1) indicated that the aromatic phenol side chain of Tyr9 protruded into the cavity of the B pocket, which may cause steric hindrance if the myristoyl group of lipopeptide ligands penetrates deeply into the B pocket (Fig. 3, *C* and *F*). These lines of structural evidence support the initial hypothesis that Ser9, rather than the Tyr9 residue, is more favorable for lipopeptide binding.

### The B pocket of HLA-A∗24:02 was reorganized upon peptide and lipopeptide binding, whereas no significant alterations were observed for the A and F pockets

**Figure 4:**
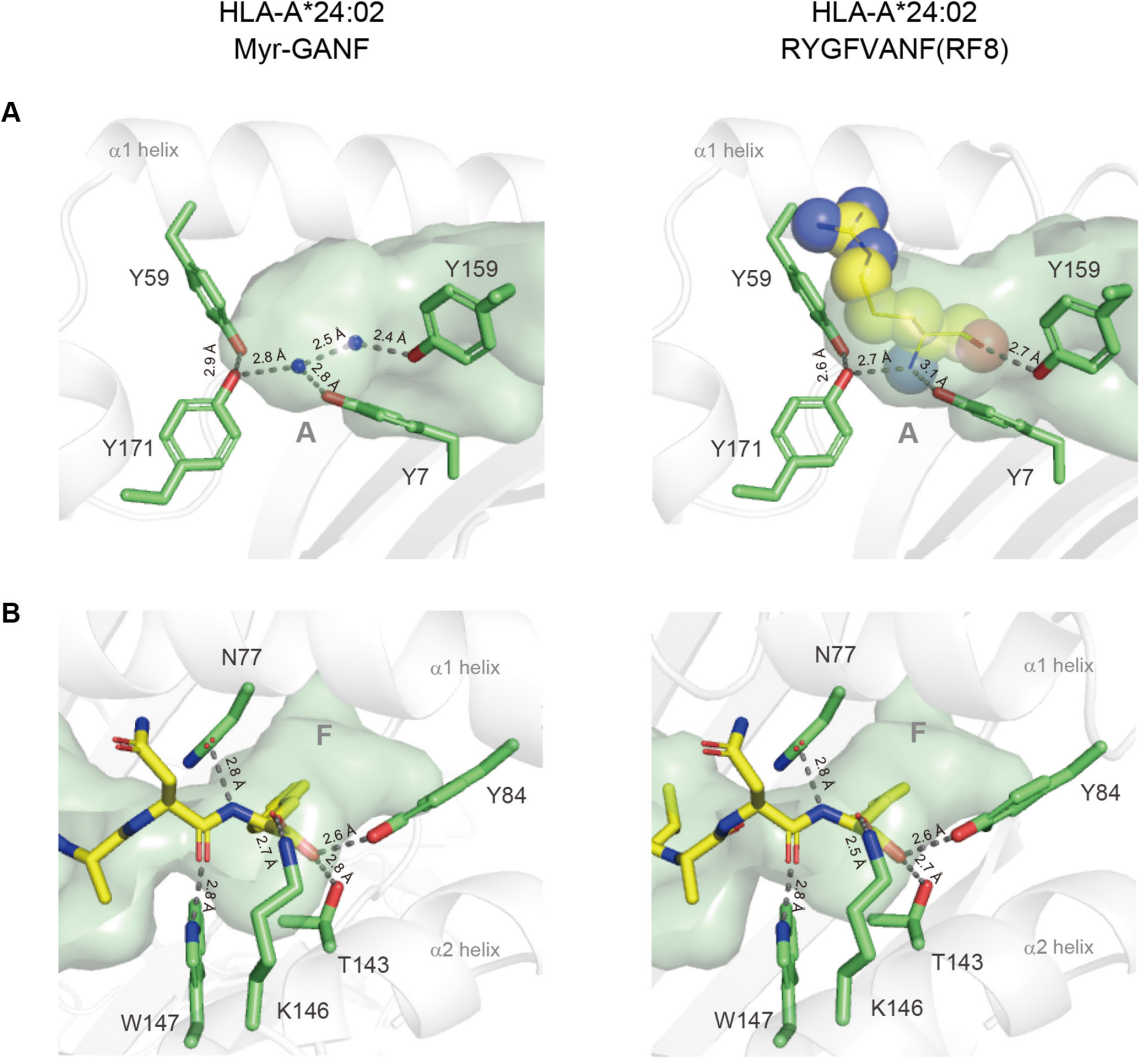
**Structures of the A and F pockets constructed in the lipopeptide-bound and peptide-bound HLA-A∗24:02 complexes.***A*, the semitransparent A pocket cavity of HLA-A∗24:02 complexed with either Myr-GANF (*left panel*) or RF8 (*right panel*) as well as side chains of a conserved cluster of tyrosine residues (*green sticks*) are shown. Note that the hydrogen-bond network (*dashed lines*) constructed in the empty A pocket using water molecules (left, indicated with *small blue spheres*) is similar to that in the P1 arginine residue (displayed as a space-filling model) containing A pocket (*right*). *B*, the semitransparent F pocket cavity of the Myr-GANF-bound (*left panel*) or RF8-bound (*right panel*) HLA-A∗24:02 complexes as well as side chains of a group of amino acid residues (*green sticks*) that establish hydrogen bonds (*dashed lines*) with the C-terminal phenylalanine residue (*yellow sticks*) are shown. HLA, human leukocyte antigen; Myr-GANF, N-myristoylated Gly-Ala-Asn-Phe.

The results obtained previously pointed to the marked ability of HLA-A∗24:02 to bind both peptide and lipopeptide ligands. To gain structural insight into this dual binding potential, the crystal structure of HLA-A∗24:02 that was complexed with the RYGFVANF (RF8) peptide (14) was also determined and compared with that of the lipopeptide-bound form (Table 1). We found that, in the peptide-bound complex, the A pocket accommodated the N-terminal Arg residue by establishing a hydrogen-bond network involving a conserved set of tyrosine residues (Tyr7, Tyr59, Tyr159, and Tyr171) with the amide group and peptide bond of the N-terminal residue (Fig. 4*A*, *right panel*). In the lipopeptide-bound complex, the A pocket was ligand-free, but the hydrogen-bond network was still maintained by the use of intervening water molecules (Fig. 4*A*, *left panel*). We also noted that the C-terminal Phe residue of the RF8 peptide was accommodated in the F pocket in a manner that was virtually identical to that for the C-terminal Phe residue of the Myr-GANF lipopeptide. In both cases, the peptide bond and carboxyl group of the C-terminal residue were stabilized by interacting with surrounding amino acid residues, Asn77, Tyr84, Thr143, Lys146, and Trp147, while the side chain anchored deeply at the F pocket (Fig. 4*B*).

**Figure 5:**
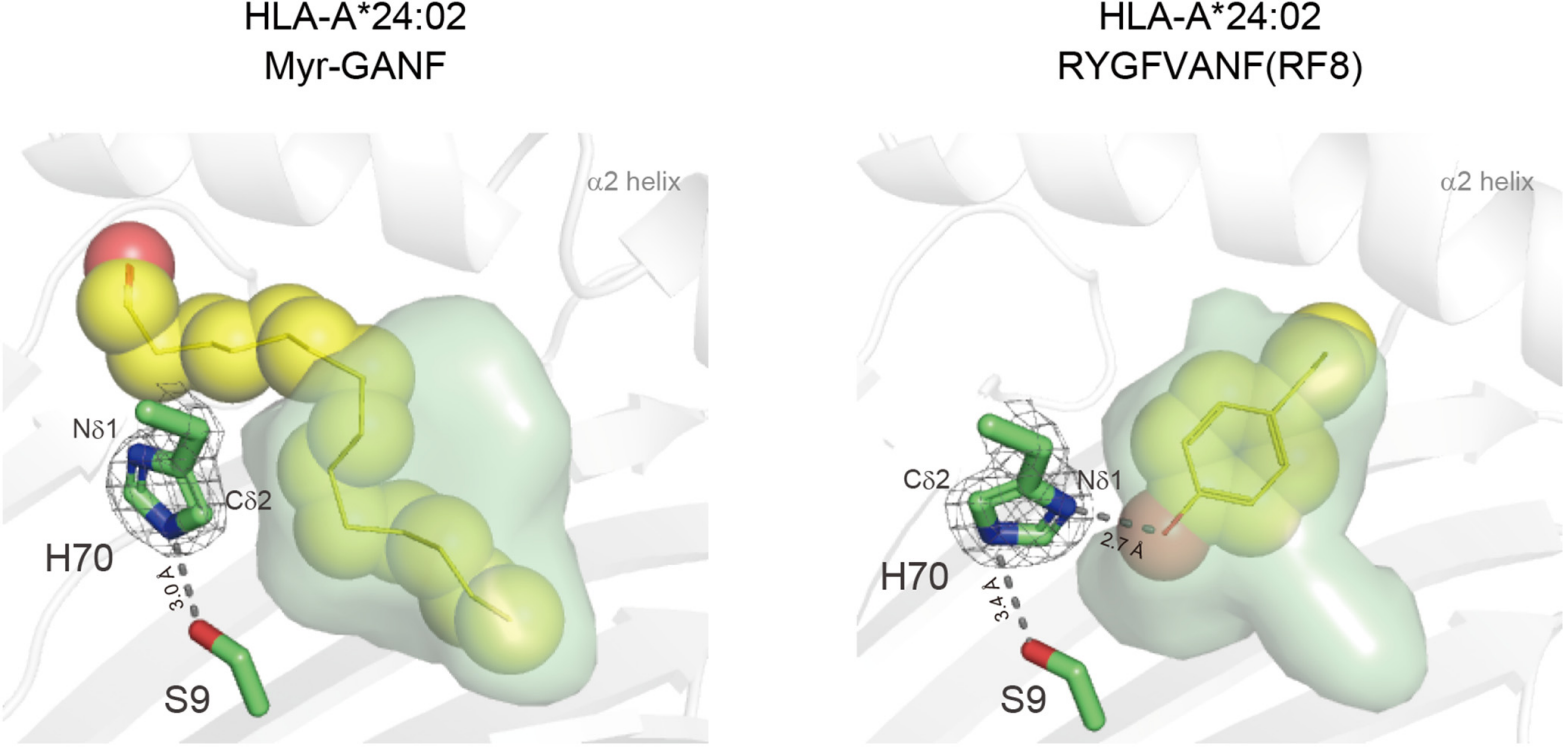
**Rotary shifts of the imidazole ring of His70 in lipopeptide-bound and peptide-bound HLA-A∗24:02 complexes.** The semitransparent B-pocket structure of the Myr-GANF-bound (*left panel*) and RF8-bound (*right panel*) HLA-A∗24:02 complexes is shown with anchor components (the myristoyl group of Myr-GANF and the side chain of the P2 tyrosine residue of RF8, respectively) depicted as space-filling models. The side chain of His70 (*green sticks*) is demonstrated by a 2Fo-Fc map (*gray mesh*) contoured at 2.0σ. Hydrogen-bond interactions (*dashed lines*) of His70 with the side chain of Ser9 (*green sticks*) and with the side chain of the P2 tyrosine residue are also depicted. HLA, human leukocyte antigen; Myr-GANF, N-myristoylated Gly-Ala-Asn-Phe.

The B pocket of HLA-A∗24:02 was able to accommodate either the long-chain fatty acid of the Myr-GANF lipopeptide or P2 amino acid residue (Tyr) of the RF8 peptide; therefore, we predicted that its pocket structure may be adaptively reorganizable in order to capture these chemically distinct anchors. In the peptide-bound complex, the hydrogen bond between Ser9 and His70 was maintained but modified in a way that supported a clockwise rotation of the χ2 dihedral angle of His70 at approximately 130 degrees (Fig. 5). This rotary shift of the imidazole ring allowed exposure of its δ nitrogen atom (Nδ1), rather than Cδ2, to the B pocket surface, leading to the establishment of a hydrogen bond with the hydroxy group of the side chain of the P2 Tyr residue (Fig. 5, *right panel*). Taken together, these observations indicate that the unique capacity of HLA-A∗24:02 to bind peptides and lipopeptides is supported primarily by a reorganized B-pocket structure upon ligand binding.

### The dual binding ability associated with B-pocket remodeling was also noted for HLA-C∗14:02, another Ser9-containing HLA class I allomorph

**Figure 6:**
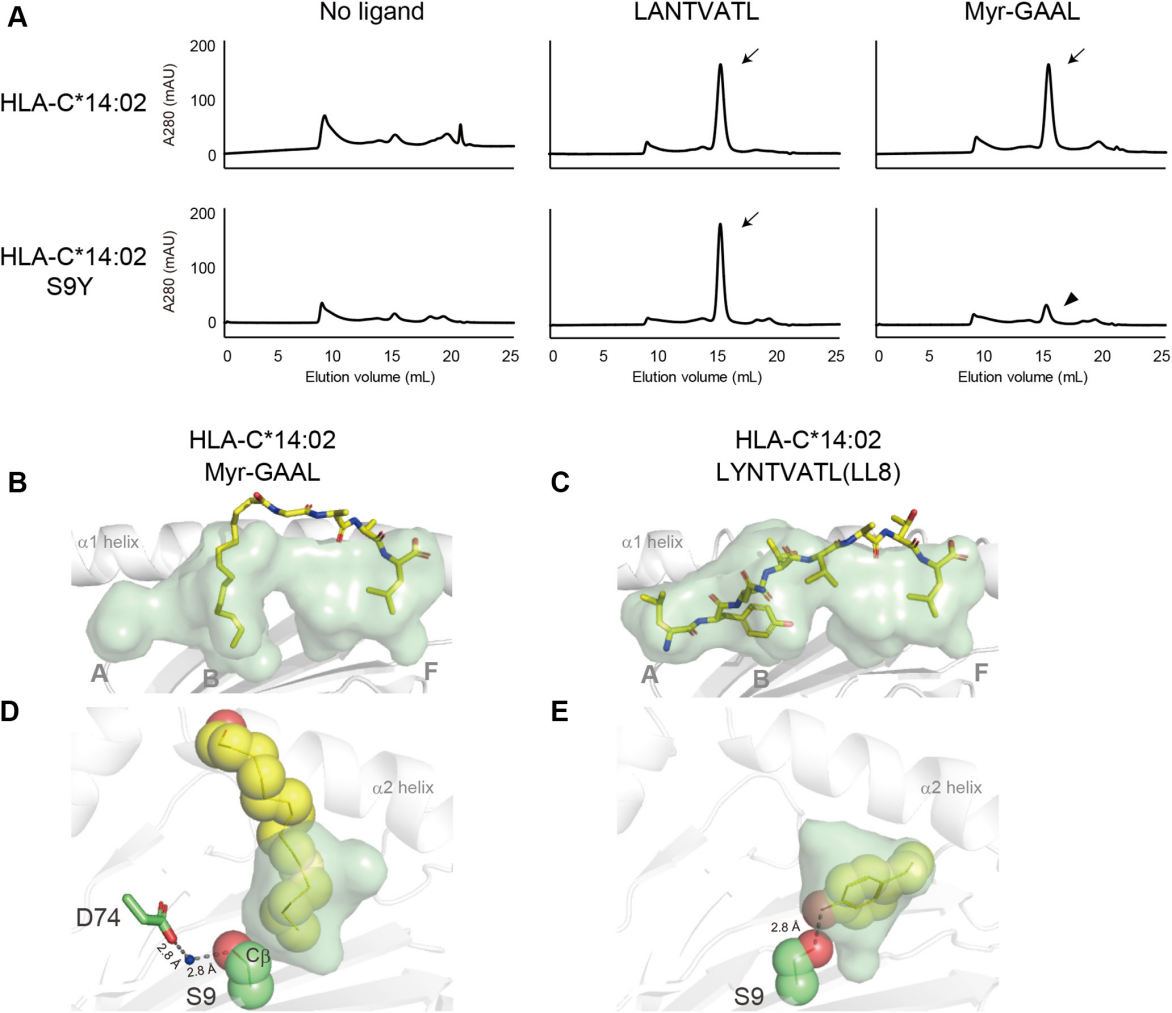
**Alterations in the B-pocket structure of HLA-C∗14:02 upon lipopeptide and peptide binding.***A*, assembly of HLA-C∗14:02 heavy chains (*upper panels*) with β2m was induced in a buffer system in the presence of either LANTVATL (*middle*) or Myr-GAAL (*right*) or in their absence (*left*), and the formation of ligand-bound HLA-C∗14:02 complexes was monitored by size-exclusion chromatography (indicated with *arrows*) as in Figure 1. Parallel experiments were performed using the S9Y mutant of HLA-C∗14:02 (*lower panels*), which detected its reduced ability to bind Myr-GAAL (*lower right* panel, indicated with an *arrowhead*). *B*–*E*, crystal structures of HLA-C∗14:02 complexed with either Myr-GAAL *(B, D)* or LYNTVATL (LL8) (*C*, *E*) were determined. The Myr-GAAL *(B)* and LL8 *(C)* ligands (*yellow sticks*) were accommodated in the semitransparent antigen-binding groove of HLA-C∗14:02. The myristoyl group of Myr-GAAL (depicted in *yellow* as a space-filling model) was captured in the semitransparent B pocket (D), whereas the P2 tyrosine residue of LL8 (depicted in *yellow* as a space-filling model) anchored at the B pocket *(E)*. *Dashed lines* indicate hydrogen bonds involving Ser9. β2m, β2-microglobulin; HLA, human leukocyte antigen.

Ser9-containing allomorphs comprise a small subset of HLA class I molecules, and no allomorphs that belong to the HLA-B family have been reported to express Ser9; however, some of the HLA-C family, including HLA-C∗14:02, contain Ser9; therefore, we examined whether the unique features observed previously for HLA-A∗24:02 may be shared with HLA-C∗14:02. Peptide-induced and lipopeptide-induced complex formation in a buffer system was observed for HLA-C∗14:02 (Fig. 6*A*, *upper panels*), whereas lipopeptide-induced, but not peptide-induced, complex formation was affected by the Ser to Tyr (S9Y) mutation at position 9 (*lower panels*). An X-ray crystallographic analysis of the lipopeptide-bound HLA-C∗14:02 complex (Table 1) revealed that the myristoyl group and C-terminal amino acid residue (Leu) of the lipopeptide ligand were accommodated in the B and F pockets, respectively (Fig. 6*B*). The hydroxyl group of the side chain of Ser9 was positioned away from the B pocket cavity by establishing a hydrogen bond with Asp74, thereby contributing to the creation of an ample cavity with a hydrophobic surface, suitable for accommodating the myristoyl group (Fig. 6*D*). These observations confirmed that the basic structural principle for lipopeptide binding was shared between HLA-A∗24:02 and HLA-C∗14:02. We also elucidated the crystal structure of HLA-C∗14:02 complexed with the LYNTVATL (LL8) peptide (15) to address whether B-pocket remodeling may occur in order to capture the P2 amino acid residue, Tyr (Table 1). We found that the hydroxymethyl side chain of Ser9 switched its spatial orientation and protruded into the B pocket cavity by disconnecting the hydrogen bond with Asp74. Alternatively, the side chain of Ser9 formed a hydrogen bond with the phenolic hydroxyl group of the P2 Tyr residue, allowing its anchoring at the B pocket (Fig. 6, *C* and *E*). Thus, we concluded that the remodeled hydrogen network involving Ser9 was fundamental for HLA-A∗24:02 and HLA-C∗14:02 to bind peptides and lipopeptides.

## Discussion

Crystal structures of peptide-bound MHC class I complexes have been studied extensively over the past 3 decades, advancing our understanding of how peptide ligands interact with the antigen-binding groove. Because the B pocket structure at which the P2 amino acid residue anchors is highly variable among MHC class I molecules, it critically regulates the repertoire of ligands that each MHC class I protein binds, as exemplified by HLA-B27 and HLA-B44 with strong preferences for arginine and glutamic acid, respectively, at the P2 position of peptide ligands (16, 17). However, the capacity of the B pocket of HLA class I molecules to accommodate nonamino acid anchors has never been explored. The present study provided structural evidence to show for the first time that B pockets of HLA-A∗24:02 and HLA-C∗14:02 are capable of binding the long-chain fatty acid component of lipopeptide ligands.

**Figure 7:**
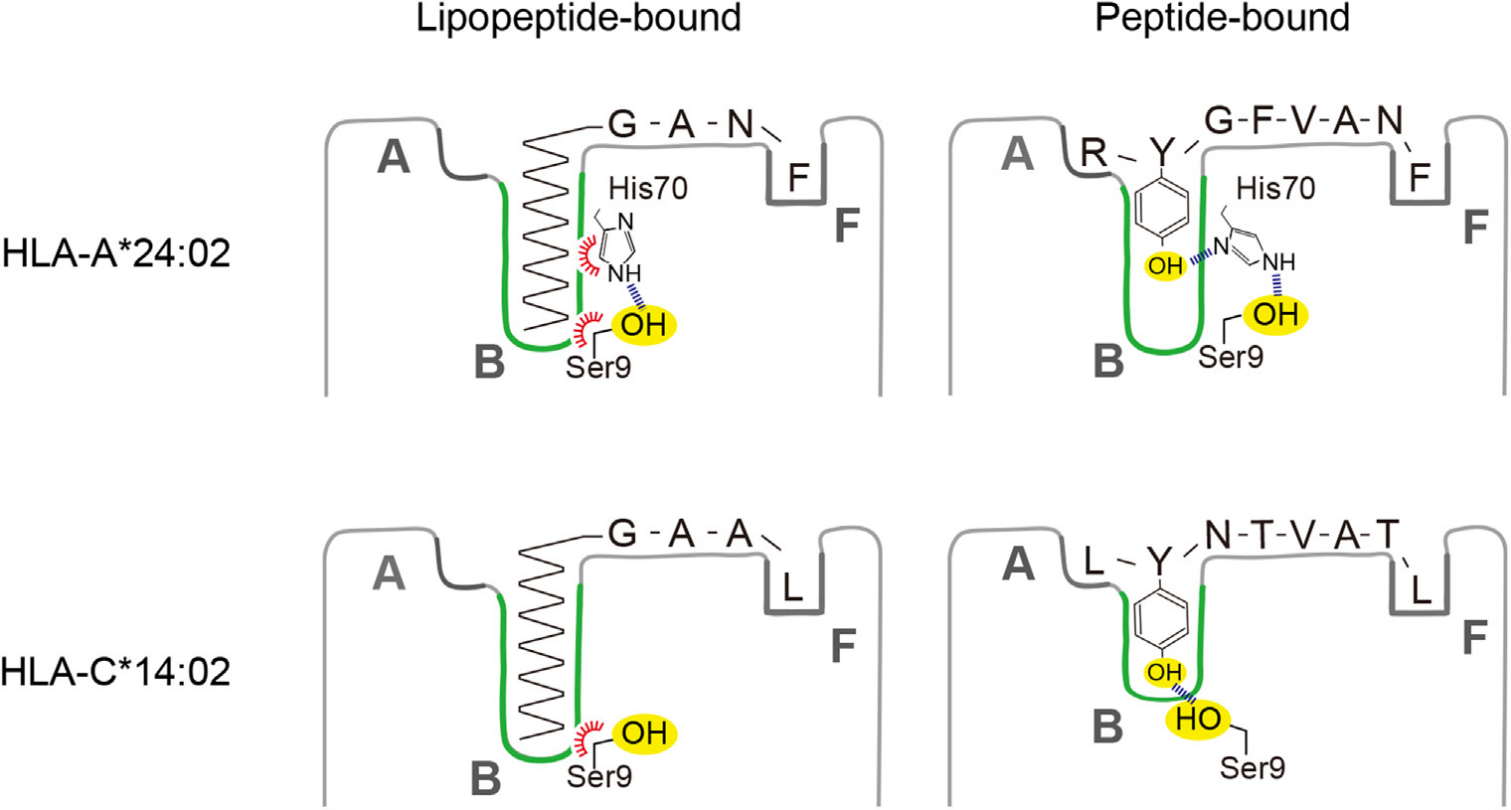
**Molecular models for the dual binding ability of HLA-A∗24:02 and HLA-C∗14:02 molecules.** The lipopeptide-bound and peptide-bound complexes of HLA-A∗24:02 (*upper panels*) as well as those of HLA-C∗14:02 (*lower panels*) are illustrated with an emphasis of the critical role of Ser9 and His70 in ligand binding. Hydrogen-bond interactions are indicated with *dotted lines*, whereas the local hydrophobic surface of the B-pocket cavity is depicted with *red arcs*. HLA, human leukocyte antigen.

As illustrated in the *left panels* of Figure 7, both allomorphs expressed Ser9 with its side chain oriented away from the B pocket cavity. This spatial arrangement of the hydroxymethyl group contributed not only to maximizing the depth of the B pocket but also to making the Cβ atom available for VDW interactions (indicated with *red arcs*). Given that these structural features are also shared with the prototypic Mamu-B∗098 (Fig. 3), we propose that the expression of Ser9 and its side-chain orientation may serve as a key element for accommodation of the long-chain fatty acid. On the other hand, this unique B pocket structure that was optimized for lipopeptide binding, was slightly but efficiently remodeled upon peptide binding in order to accommodate the P2 amino acid residue. In the case of HLA-C∗14:02, a direct strategy was adopted in which the hydroxymethyl side chain of Ser9 was redirected toward the B-pocket cavity. This resulted in raising the bottom of the B pocket and increasing the surface hydrophilicity, thereby successfully creating a hydrogen bond with the P2 amino acid residue (Fig. 7, *lower panels*). Thus, the hinged switch-like movement of the hydroxymethyl group of Ser9 appeared to control peptide and lipopeptide binding. Alternatively, HLA-A∗24:02 adopted a rather indirect strategy in which spatial shifts were induced not for Ser9 itself but for its hydrogen bond partner, His70. Upon lipopeptide binding, the Cδ2 atom of the imidazole ring of His70, as well as the Cβ atom of Ser9, is exposed to the pocket cavity to achieve VDW interactions with the myristoyl group (Fig. 7, *upper left panel*, indicated with *red arcs*). In contrast, rotation of the imidazole ring was induced upon peptide binding, resulting in exposure of the Nδ1 atom to the pocket surface to hydrogen bond with the P2 amino acid residue (*upper right panel*). Therefore, specific amino acid residues other than Ser9 also control lipopeptide binding, suggesting that some but not all Ser9-containing HLA class I allomorphs may have evolved lipopeptide-binding ability.

Viruses utilize host-derived N-myristoyltransferase and myristoyl-CoA to achieve N-myristoylation of their own proteins, and N-myristoylated viral proteins often dictate their pathogenesis (18). Therefore, the expansion of the repertoire of MHC class I–bound ligands that includes lipopeptides besides conventional peptides may confer advantages in evolutionary terms. As stated previously, none of the HLA-B molecules expresses Ser9 while Ser9-containing allomorphs comprise 19% and 14% in the HLA-A and HLA-C families, respectively. In rhesus monkeys, 43% of Mamu-B alleles encode Ser9-containing allomorphs, comprising a major subset in this family, while only 7% of Mamu-A1 allomorphs express Ser9. Thus, it is interesting to consider that each vertebrate is still undergoing evolutionary processes to create allomorphs that have evolved the ability to bind both peptides and lipopeptides and that efficiencies in this task vary significantly among MHC class I loci of individual species (19). In this respect, Mamu-B∗098 may be regarded as an extreme example in that it has lost the ability to bind peptides by occluding the channel connecting between the A and B pockets while maintaining the ability to bind lipopeptides (5, 20).

In uninfected, healthy cells, only a tiny fraction of cellular proteins is N-myristoylated (7). Accordingly, cellular peptides are likely to predominate over lipopeptides as endogenous ligands for HLA-A∗24:02 and HLA-C∗14:02. Shortly after virus invasion into the cells, however, dysregulated production of N-myristoylated viral proteins may occur, which is associated with the accumulation of N-terminal lipopeptide fragments derived from defective ribosomal products or DRiPs (21). Under such pathological conditions, the transient and rapid increase in the cellular pool of viral lipopeptides may allow HLA-A∗24:02 and HLA-C∗14:02 to sample lipopeptide ligands efficiently and activate lipopeptide-specific CTLs.

The ability of these HLA class I molecules to present lipopeptides to T cells remains to be addressed, which depends on whether potential T-cell epitopes are exposed for T-cell recognition and whether the repertoire of functional T-cell receptors (TCRs) are prepared. An X-ray crystallographic analysis of lipopeptide-bound Mamu-B∗05104 in a form that is cocrystallized with specific TCRs indicates that the amide group of the N-myristoylated glycine residue (Myr-Gly1) hydrogen bonds with the TCR CDR3β loop, thus offering a primary T-cell epitope (22). On the other hand, TCRs are positioned remotely from the peptide portion of lipopeptide ligands, and no direct interactions are observed, suggesting that lipopeptides are recognized in a way distinct from the well-established mechanism for peptide recognition. Because the spatial orientation of the amide group of Myr-Gly1 differs significantly among N-myristoylated lipopeptides, we hypothesize that the peptide portion of lipopeptide may control antigenicity indirectly rather than offering primary T-cell epitopes.

The lipopeptide-binding capacity of HLA class I molecules demonstrated in the present study sheds light on a new aspect of the MHC class I system that has evolutionary and medical implications.

## Experimental procedures

### Synthesis of peptide and lipopeptide ligands

N-myristoylated 4-mer lipopeptides, Myr-GANF and Myr-GAAL, as well as 8-mer peptides, LANTVATL and LYNTVATL (LL8), were synthesized by manual Fmoc solid-phase peptide synthesis using Wang resin preloaded with either phenylalanine (for Myr-GANF) or leucine (for the other compounds) (Watanabe Chemical Industries). Acylation was carried out by reacting the N-terminal amide group with myristic acid anhydrides, as described previously (10). The synthesized products were released in 95% TFA and prepared as a form of acetate salts. The 8-mer peptides RAGFVANF and RYGFVANF (RF8) were purchased from GenScript Japan Inc.

### Protein refolding and size-exclusion chromatography

Complementary DNA sequences encoding the ectodomains (from Gly-1 to Pro-276) of HLA-A∗24:02 and HLA-C∗14:02 were synthesized (IDT Inc) and cloned into pET21c(+) (Addgene). Complementary DNA constructs encoding HLA-A∗24:50 and the S9Y mutant of HLA-C∗14:02 were generated by site-directed mutagenesis. The expression plasmids were introduced into the *Escherichia coli* Rosetta 2 (DE3) pLysS strain (Novagen), and recombinant proteins were expressed as inclusion bodies. To obtain HLA-A∗24:02 and HLA-C∗14:02 complexes, purified heavy chains (1 mmol/L) and β2m (1 mmol/L) were assembled by rapid dilution in a refolding buffer in the presence of a 20-fold molar excess of specific ligands, as described previously (5). Each sample was then dialyzed against 10 mM Tris–HCl, pH 8.0, and subjected to size-exclusion chromatography using the Superdex 200 Increase column (10 × 300 mm, GE Healthcare) at a flow rate of 0.8 ml/min. The formation of the heavy chain–β2m–ligand trimer complexes was monitored by an increase of *A*_280_ values at an elution volume of 15 ml.

### Crystallization and structure determination

The ligand-bound HLA class I complexes were purified sequentially by size-exclusion chromatography, followed by monoQ (GE Healthcare) anion exchange chromatography, and crystals were formed by a sitting-drop vapor-diffusion method. For the HLA-A∗24:02:Myr-GANF complex, 1 μl of protein solution (7 mg/ml in 50 mM NaCl, 10 mM Tris, pH 8.0) was mixed with 1 μl of a mother liquid containing 2 mM ZnCl2, 100 mM Tris, pH 8.0, and 20% PEG 6000 and incubated at 4 °C. For the HLA-A∗24:02:RF8 complex, 1 μl of protein solution (10 mg/ml in 50 mM NaCl, 0.1 mM ZnCl2, 10 mM Tris, pH 8.0) was mixed with 1 μl of a mother liquid containing 100 mM Tris, pH 7.8, and 20% PEG 6000 and incubated at 20 °C. For the HLA-A∗24:50–RAGFVANF complex, 1 μl of protein solution (10 mg/ml in 50 mM NaCl, 0.1 mM ZnCl2, 10 mM Tris, pH 8.0) was mixed with 1 μl of a mother liquid containing 100 mM Tris, pH 7.8, and 20% PEG 8000 and incubated at 4 °C. For the HLA-C∗14:02–Myr-GAAL complex, 1 μl of protein solution (10 mg/ml in 10 mM Tris, pH 8.0) was mixed with 1 μl of a mother liquid containing 200 mM ammonium acetate, 100 mM sodium acetate, pH 4.7, 25% PEG Smear Low (Molecular Dimensions), and 5% ethylene glycol and incubated at 4 °C. For the HLA-C∗14:02–LL8 complex, 1 μl of protein solution (10 mg/ml in 10 mM Tris, pH 8.0) was mixed with 1 μl of a mother liquid containing 100 mM sodium acetate, pH 4.5, and 20% PEG Smear Low and incubated at 20 °C. The crystals were cryoprotected in 20% ethylene glycol, and diffraction data were collected at 100 K (in a cold nitrogen gas stream) either on an MX-225HE detector (Rayonix) or on an EIGER X 4M detector (DECTRIS) with a wavelength of 1.0 Å. The resulting datasets were processed, merged, and scaled using XDS (Max Planck Institute for Medical Research) for HLA-A∗24:02 and HKL-2000 (HKL Research) for HLA-C∗14:02. Structures of the ligand-bound HLA-A∗24:02 and HLA-A∗24:50 complexes were clarified by molecular replacement with the HLA-A∗24:02 complex (Protein Data Bank ID: 4F7T) as a search model, by MOLREP as implemented in CCP4i software (http://legacy.ccp4.ac.uk/index.php), whereas those of the ligand-bound HLA-C∗14:02 complexes were resolved by MOLREP with the HLA-C∗06:02 complex (Protein Data Bank ID: 5W6A) as a search model. The models were refined using REFMAC5 and PHENIX1.9/1.10 software (http://www.phenix-online.org). The structures were rebuilt using COOT 0.8.9 and further modified based on σ-weighted (2|Fo|—|Fc|) and (|Fo|—|Fc|) electron density maps. Crystallographic images were created using PyMOL software (DeLano Scientific).

## Data availability

All data contained within the article.

## Conflict of interest

The authors declare that they have no conflicts of interest regarding the contents of this article.
